# Potential Metabolic Pathways Involved in Osteoporosis and Evaluation of Fracture Risk in Individuals with Diabetes

**DOI:** 10.1155/2024/6640796

**Published:** 2024-05-23

**Authors:** Tong Liu, Yanjun Wang, Bing Qian, Pan Li

**Affiliations:** Emergency Department Honghui Hospital Xi'an Jiaotong University School of Medicine, Xi'an, China

## Abstract

Diabetes has a significant global prevalence. Chronic hyperglycemia affects multiple organs and tissues, including bones. A large number of diabetic patients develop osteoporosis; however, the precise relationship between diabetes and osteoporosis remains incompletely elucidated. The activation of the AGE-RAGE signaling pathway hinders the differentiation of osteoblasts and weakens the process of bone formation due to the presence of advanced glycation end products. High glucose environment can induce ferroptosis of osteoblasts and then develop osteoporosis. Hyperglycemia also suppresses the secretion of sex hormones, and the reduction of testosterone is difficult to effectively maintain bone mineral density. As diabetes therapy, thiazolidinediones control blood glucose by activating PPAR-*γ*. Activated PPAR-*γ* can promote osteoclast differentiation and regulate osteoblast function, triggering osteoporosis. The effects of metformin and insulin on bone are currently controversial. Currently, there are no appropriate tools available for assessing the risk of fractures in diabetic patients, despite the fact that the occurrence of osteoporotic fractures is considerably greater in diabetic individuals compared to those without diabetes. Further improving the inclusion criteria of FRAX risk factors and clarifying the early occurrence of osteoporosis sites unique to diabetic patients may be an effective way to diagnose and treat diabetic osteoporosis and reduce the risk of fracture occurrence.

## 1. Introduction

Diabetes mellitus is a group of multietiological metabolic diseases characterized by chronic hyperglycemia due to deficiencies in insulin secretion and/or utilization [1]. About 537 million people aged 20-79 years had diabetes worldwide in 2021. By 2045, that number could reach 783 million [2]. Osteoporosis is another disease with a high incidence in the population, which can be divided into primary and secondary causes. It is characterized by decreased bone mass and damage to the microstructure of bone tissue, leading to increased bone fragility and susceptibility to fracture. Primary osteoporosis is common in postmenopausal women and the elderly, while secondary osteoporosis is often caused by endocrine and metabolic diseases or systemic diseases, such as hyperparathyroidism and diabetes. Fractures often occur in people with osteoporosis as a result of light activity, weight bearing, or a fall. Most of the fracture sites are hip, spine, and forearm. Therefore, for patients with diabetes or osteoporosis, how to improve the symptoms and prevent further deterioration of the disease or even fracture is extremely important. This paper will summarize the partially related mechanisms of diabetes and osteoporosis based on existing studies, summarize the effects of common hypoglycemic drugs on bone, and review the fracture risk and assessment tools of diabetic patients.

## 2. Mechanism of the Interaction between Diabetes Mellitus and Osteoporosis

Both type 1 diabetes and type 2 diabetes have a higher incidence of osteoporosis and fragility fractures than nondiabetic patients [3, 4]. However, the mechanism of osteoporosis in diabetic patients is not fully understood. Therefore, the ways and ways that diabetes affects bone metabolism have become an urgent problem to be solved.

Diabetes mellitus is often divided into type 1 diabetes mellitus and type 2 diabetes mellitus, and the mechanism of action of the two diabetes mellitus is not the same. Type 1 diabetes is characterized by the destruction of pancreatic islet *β*-cells and an absolute insulin deficiency [5]. Type 2 diabetes is characterized by insufficient insulin secretion and insulin resistance, which is the reduced sensitivity of target organs to insulin action [6]. The liver is one of the main target organs of insulin, and the synthesis of hepatic glycogen plays an important role in the relative stability of blood glucose levels in the body. Moreover, insulin signaling in skeletal muscle and adipose tissue also has an indispensable role on the regulation of blood glucose levels.

Diabetes mellitus is characterized by hyperglycemia. The main role of insulin is to reduce blood sugar levels and maintain glucose homeostasis in the body. In islet *β* cells, proinsulin is first synthesized, and in the process, it is converted to proinsulin through the endoplasmic reticulum, transfers to the Golgi, enters the immature secretory vesicles, and eventually cleaves into C peptide and insulin [7–9]. Performing this process requires ensuring the integrity of islet *β*-cells and the strict regulation of their function [10]. Therefore, both *β* disruption and dysfunction of the cells may lead to deficient secretion of insulin synthesis.

Insulin receptors are abundantly present on the liver cell membrane and become phosphorylated when bound to insulin, activating downstream signaling factors such as IRS 2 [11]. Upon phosphorylation of IRS 2, PI3K is activated. Therefore, insulin activates PI3K/AKT by stimulating the insulin receptor, which subsequently promotes cellular glucose uptake, reduces glucose levels in plasma, and maintains glucose homeostasis [12, 13]. The PI3K/AKT signaling pathway plays an important regulatory role in protein and glycogen synthesis as well as cellular senescence and death [14, 15]. This pathway can downregulate TXNIP, avoid the latter overexpression of pancreatic *β*-cells apoptosis, and then reduce tissue sensitivity to insulin [16, 17]. Activation of PI3K/AKT signaling upregulates GLUT 4 expression, which in turn promotes glucose entry into cells for glycolysis and suppresses gluconeogenesis in the liver, while inhibition of this pathway leads to insulin resistance [18, 19]. Insulin resistance is associated with the occurrence of many diseases, perhaps due to the restricted glucose uptake interfering with the cellular energy supply [20].

Bone changes with two processes: bone modeling and bone remodeling [21]. Reducing bone remodeling or an imbalance between bone formation and absorption can lead to bone diseases such as osteoporosis [22]. One of the causes of osteoporosis is diabetes mellitus. Osteoblasts versus osteoclast activity can be regulated by insulin signaling, leading to the regulation of bone formation and bone resorption activity [23, 24]. In insulin deficiency, osteoblast numbers decrease, mineral deposition rate decreases, and bone integrity is impaired.

### 2.1. Advanced Glycosylation End Products

There are several theories about the reasons why diabetes is harmful to bone, such as the accumulation of AGEs due to poor blood glucose control, which increases the production of nonenzyme crosslinkers in collagen fibers, thus negatively affecting bone matrix properties and affecting bone reconstruction, and directly activating inflammatory and oxidative stress pathways to promote osteoporosis.

Advanced glycosylation end products are formed by protein glycosylation. Nonenzymatic reaction between ketones or aldehydes and protein amino groups facilitates abnormal protein function. This process can occur in both the normal and high-glucose states [25, 26]. This process proceeds from the Schiff base adduct to the Amadori rearrangement product and finally to the Maillard reaction, eventually forming irreversible advanced glycosylation end products [27, 28]. The speed of the glycosylation process depends on the availability of the substrate. Exogenous advanced glycosylation end products can be formed in hot-processed foods, and endogenous advanced glycosylation end products can be formed by the ingestion of large amounts of sugar [29]. Normal human blood glucose levels are stable, and the production of AGEs is moderate. Diabetic patients have higher blood glucose levels and increased glucose availability, leading to a significantly accelerated production of AGEs [30]. Excess glucose is reduced to sorbitol by aldol reductase and then converted to fructose by sorbitol dehydrogenase. Fructose and its metabolites are potent glycosylating agents that can lead to the production of AGEs.

Advanced glycosylation end products are a group of destruction-competent compounds that gradually form in the high-glucose state and are widespread in the extracellular matrix. The late glycosylation end products are not only extracellular matrix proteins, but also intracellular proteins. Advanced glycation end products interact with these proteins, impairing the function of the latter group. Moreover, the binding of late glycosylated terminal products to the late terminal products receptor is also a classical pathway to function. The combination of the two can lead to many types of diabetic complications, in which the inhibition of BMSC function reduces osteogenic differentiation, followed by osteoporosis [31, 32]. Sun et al. showed that activation of AGE-RAGE signaling caused inhibition of osteogenic differentiation, while both bone-associated genes and proteins were significantly enhanced after inhibiting RAGE [33]. The negative effects of AGE-RAGE signaling on BMSCs can be suppressed by increasing the expression of Glo-1. The receptor for advanced glycosylation end products belongs to the multiligand receptor, which can bind to advanced glycosylation end products, Mac-1, high-mobility group protein 1, and S100 calcium granulin [34–36]. In vitro experiments show that binding of late glycation end products to its receptor activates p38 mitogen-activated protein kinase, extracellular signal-regulated kinase 1/2, Janus kinase, and Rho-GTP enzymes [37–40]. Moreover, after the binding of the late glycosylation terminal product receptor to the ligand, the ligand activates the NADPH oxidase, which increases the formation of reactive oxygen species in the cell [41, 42]. Reactive oxygen species can not only promote the formation of advanced glycation end products and further aggravate the damage to the body but also activate the transcription factor nuclear factor kappa B, increase the expression of proinflammatory factors, and then aggravate the inflammatory response of the body. Both oxidative stress and an increased inflammatory response can lead to the dysfunction of osteoblasts.

In addition to functioning by binding to extracellular RAGE, late glycation end products can also modify intracellular proteins for osteoblast apoptosis. Accumulation of AGEs in cells leads to ER stress, which, in the short term, promotes the secretion of proinflammatory cytokines and attracts immune cells, thus upregulating the expression of GLUT 1 and GLUT 3, while persistent ER stress leads to chronic inflammation or tissue damage, such as osteoporosis. Related studies have also confirmed that the intracellular accumulation of AGEs can induce apoptosis in osteoblasts [43]. Moreover, ER stress also increases *β*-amyloid production, which further aggravates insulin resistance.

Ge et al. showed a significant inhibitory effect of advanced glycation end products on the proliferation and differentiation of osteoblasts [44]. Further studies suggested that advanced glycation end products increase the occurrence of iron death within osteoblasts. A series of consequences of iron death will be described later.

### 2.2. Iron Metabolism

Iron-dependent lipid peroxidation causes ferroptosis, and iron metabolism disorders have an influence on osteoporosis [45, 46]. At the same time, it is reported that glucose and lipid metabolism affects ferroptosis to some extent [47, 48]. Serum iron and ferritin were significantly higher in diabetic rats, while the expression of Gpx 4 was significantly reduced. As one of the markers of ferroptosis, Gpx 4 is widely expressed in bone tissue, and its expression level was decreased significantly after high glucose treatment, and it was increased significantly after the addition of ferrostatin-1, which also confirmed that high glucose environment can trigger ferroptosis [49]. Further studies suggested that high glucose-induced ferroptosis is most likely mediated through the METTL3/ASK 1 pathway in diabetic rats. And knockdown of METTL3 alleviated high glucose-induced osteogenic dysfunction [47]. Thus, diabetes can lead to osteoporosis by inducing ferroptosis. Then, improving or inhibiting ferroptosis can protect the bone and delay or avoid the occurrence of osteoporosis. Studies have shown that the daily intake of a certain dose of vitamin K2 reduces the risk of diabetes [50]. Interestingly, vitamin K cycling significantly inhibited ferroptosis [51]. Vitamin K2 can function through the AMPK/SIRT 1 signaling pathway. Studies have shown that high glucose environment can induce ferroptosis in BMSCs and osteoblasts, and ferrostatin-1 (Fer-1) can inhibit ferroptosis, restore the survival of BMSCs in high glucose environment, and reverse the inhibitory effect of high glucose on osteoblasts. The study showed that vitamin K2 also alleviated mitochondrial dysfunction and ferroptosis in BMSCs in high glucose. Its in vitro experiments proved that vitamin K2 can also combat the inhibition of osteogenic differentiation by high glucose, and the expression of RUNX 2 increased significantly with vitamin K2 treatment. Further studies revealed that SIRT 1 knockout significantly decreased RUNX 2 levels in cells treated with vitamin K2. The protective effect of vitamin K2 on BMSCs is accomplished through the AMPK/SIRT 1 signaling pathway. By protecting MSC function, vitamin K2 can have a beneficial effect on the prevention or treatment of osteoporosis in diabetic patients [52].

### 2.3. Sex Hormone

**Figure 1:**
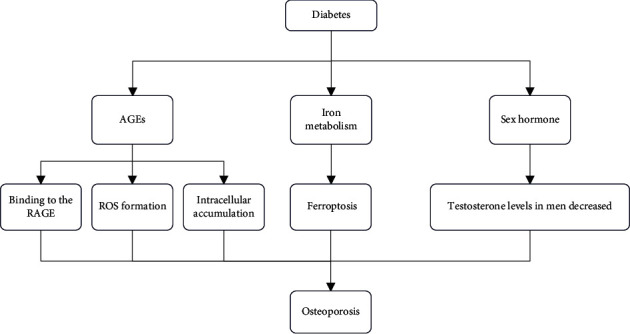
The AGEs were increased in diabetic patients. AGEs inhibit osteoblast differentiation after binding to RAGE. RAGE binding to ligand increases ROS formation, leading to osteoblast dysfunction. Intracellular accumulation of AGEs can induce apoptosis in osteoblasts. The high glucose environment aggravates ferroptosis, leading to osteoblast dysfunction. Male diabetic patients have decreased testosterone levels. Decreased testosterone levels increase bone turnover, leading to bone loss. The above process eventually leads to osteoporosis.

Chronic hyperglycemia plays an inhibitory effect on the hypothalamic-pituitary-gonadal axis [53]. In bone metabolism processes, sex hormones play an important role. There is no consensus on the effect of androgens on blood glucose. Studies have reported that a large number of patients with type 2 diabetes will develop decreased testosterone [54]. It is also believed that a lower testosterone level is an independent risk factor for the occurrence of type 2 diabetes, while a higher level of testosterone can have a preventive effect on type 2 diabetes [55, 56]. However, studies in women have shown that increasing total testosterone with calculated free testosterone levels is associated with a higher risk of type 2 diabetes [57]. There are theories calculated that androgen levels are associated with more severe insulin resistance, but the mechanisms are unclear [58]. There is some speculation that this is related to adipocyte dysfunction [59]. Not only the specific role and mechanism of androgens are unclear, but the relationship between estrogen and insulin resistance is not elucidated. Some studies suggest a positive relationship between estradiol and insulin resistance in women, but not in men [60–64]. Another part of the studies held the exact opposite result [65–67]. However, sex hormone-binding globulin (SHBG) was inversely associated with insulin resistance and type 2 diabetes in both men and women [68, 69]. A meta-analysis of sex hormones and type 2 diabetes showed gender differences in the association between testosterone and type 2 diabetes [56]. Testosterone levels were generally higher in women with diabetes, but significantly lower in males. Testosterone has a stimulating effect on the differentiation and proliferation of osteoblasts [70, 71]. A lack of testosterone can increase bone turnover and even bone loss [72]. Some experiments show that testosterone plays an anti-inflammatory role in several types of tissues, which could explain the pathogenesis of some type 2 diabetes and insulin resistance [73–75]. In addition, estradiol is mainly converted from testosterone. Estrogen promotes osteoclast apoptosis in vivo and inhibits osteoclast activity, thereby reducing bone resorption [76, 77]. Studies showed a negative association between bone mineral density and testosterone and a positive association with estradiol in men [78]. In addition, the level of bone turnover markers is also associated with estrogen [79, 80].  Figure 1 illustrates the possible mechanism of osteoporosis secondary to diabetes.

## 3. Effect of the Drugs on the Bone

Diabetic patients will use hypoglycemic drugs to control their blood sugar. Different kinds of hypoglycemic drugs have different effects on bone metabolism in the body. Although the use of some drugs controls blood sugar, it affects bone metabolism, leading to the emergence of osteoporosis, and even increases the risk of bone fractures.

### 3.1. Thiazolidinedione

Thiazolidinediones represented by rosiglitazone and pioglitazone are common antidiabetic drugs. The effects of thiazolidinediones on the body have been previously reported. It achieves blood glucose control effects by improving insulin sensitivity while reducing insulin resistance. But blood glucose control by thiazolidinediones is accomplished through the activation of the peroxisome proliferator-activated receptor gamma (PPAR *γ*). The PPAR *γ* protein plays an important role in glucose homeostasis and adipogenesis. After its activation, the adipogenesis increases in the body, and the bone metabolism is unbalanced. It increases the generation of osteoclasts and reduces the production of osteoblasts, resulting in enhanced bone resorption activity and reduced bone formation [3, 81–83]. Thiazolidinediones increase the transcriptional activity of PPAR-*γ* by increasing the expression of fibroblast growth factor 21 (FGF21), thereby improving insulin resistance in diabetic patients [84].

Thiazolidinediones can be used to treat diabetes, but they have other effects on bone. As mentioned above, thiazolidinedione can improve insulin resistance and improve insulin sensitivity in diabetic patients through FGF21. Indeed, FGF21 can cause a decrease in bone mass. Wei et al. showed that bone loss caused by rosiglitazone can be prevented by inhibiting the function of FGF21 [85]. In addition to FGF21, thiazolidinediones can also increase FGF1 expression by activating PPAR-*γ*, similarly avoiding the appearance of insulin resistance [86].

Existing studies show that PPAR-*γ* is mainly present in adipose tissue, but it is also expressed in liver, muscle, macrophages, etc. Lack of PPAR-*γ* or inhibition of PPAR-*γ* at different locations will cause some degree of insulin resistance [87–90].

In addition to antidiabetic effects, thiazolidinediones have another effect on bone. Many studies have reported an increased risk of fracture after taking thiazolidinediones such as rosiglitazone and pioglitazone [91–93]. Thiazolidinedione-activated PPAR-*γ* can differentiate MSCs into adipoblast lineage rather than osteoblast lineage and then differentiate into adipocytes and can also promote the differentiation of osteoclasts and the increase of osteoclast number [94–96]. This will affect the bone mineral density of patients to some extent, and the reduced bone mineral density may indeed increase the risk of fracture. But the meta-analysis by Billington et al. suggested that thiazolidinediones may influence fracture risk through other mechanisms than by reducing bone density [97]. Previous animal experiments showed that animals treated with thiazolidinediones had increased cortical porosity and decreased cortical thickness, but no significant changes in bone mineral density were observed [98, 99]. In patients taking thiazolidinediones, numerous fractures occur in cortical areas without significant more severe bone loss [91, 100]. At present, the mechanism of thiazolidinediones affecting bone and increased fracture risk is not clear, and investigations in patients with long-term use of such drugs are still needed to learn more about the benefits and risks of thiazolidinediones in patients with diabetes.

### 3.2. Metformin

Metformin, as a classic oral antidiabetic agent, works by improving insulin sensitivity. Numerous studies have shown that in addition to blood glucose control, metformin has some protective effects on bone. In vitro experiments showed that metformin can promote proliferation and differentiation of osteoblasts. In addition, metformin also reduces gluconeogenesis in the liver, thus partly avoiding the negative effects of high glucose on bone. Earlier studies showed that metformin controlled hepatic gluconeogenesis in two ways, AMPK-dependent and AMPK-independent [101]. Many previous in vitro studies have confirmed the osteogenic effect of metformin [102]. In fact, metformin can be osteogenic both in vitro and in vivo. By activating AMPK and RUNX 2, metformin promoted the differentiation of myeloid precursor cells into osteoblasts. Recently, metformin that can regulate PPAR-*γ* through the AMPK pathway to achieve a protective effect on osteoblast differentiation and thus against osteoporosis was shown by Zheng et al. [103]. Long-term poor glycemic control also allows for the accumulation of advanced glycation end products in diabetic patients. The AGEs, on the one hand, inhibit the differentiation of osteoblasts, and on the other hand, the deposition of type I collagen is also affected by the bone quality.

In addition to reducing the negative bone effects of hyperglycemia and advanced glycation product accumulation, metformin can also reduce bone loss caused by rosiglitazone, which subsequently maintains bone density in patients [104]. This may also be accomplished through the regulation of PPAR-*γ*.

In addition to its protective effect on osteoblast differentiation, metformin can also exert an inhibitory effect on osteoclast differentiation. Osteoblasts can secrete RANKL and osteoprotegerin. RANKL actively promotes during osteoclast differentiation, and osteoprotegerin, as a decoy receptor for RANKL, can reduce osteoclast differentiation. In vivo experiments showed that after metformin treatment, osteoprotegerin levels increased and RANKL expression was inhibited in rats [105]. Inhibition of osteoclast differentiation weakens bone resorption, thereby reducing bone loss.

Metformin treatment improved bone mineral density in type 2 diabetes, and this effect was more pronounced in women [106]. Animal experiments confirmed that metformin prevented bone loss by inhibiting the expression of RANKL in mice after ovariectomy [105]. Moreover, not only for diabetic patients, postmenopausal women may also benefit from metformin in preventing osteoporosis.

Although bone mineral density was not significantly reduced in type 2 diabetes, their fracture risk remains significantly higher. However, patients with metformin application not only reduce the risk of osteoporosis but also can reduce the risk of osteoporotic fractures at multiple sites, including vertebral fractures [107, 108]. This may be due to metformin preventing the bone loss and protecting the bone quality.

However, not all studies have considered metformin as beneficial for bone. According to Jeyabalan et al., metformin has no significant effect on bone mass or the healing of fracture [109].

Although most of the current studies believe that metformin is beneficial for the prevention of osteoporosis, the mechanism still needs to be further explored, and the time-dose effect study of metformin is still incomplete, which is also a direction of future research. An in-depth study of the mechanism of metformin on bone and its time-dose effect may also give more valuable guidance to clinicians.

### 3.3. Insulin

Insulin is an important therapeutic agent for patients with type 1 and type 2 diabetes mellitus. Its effects on bone are divided into direct and indirect effects, and it is still controversial.

The direct effect of insulin on the skeleton is accomplished by its interaction with the IGF-1 receptor in osteoblasts. Insulin acts similar to IGF-1 in acting with the IGF-1 receptor. Studies have shown that IGF-1 favors the accumulation of osteoblasts and reduces bone loss [110]. It is therefore likely that insulin has a positive effect on bone synthesis, maintaining or enhancing bone density. Studies have suggested that insulin deficiency is also responsible for less bone mass in patients with type 1 diabetes than in those with type 2 diabetes [111]. Insulin receptor substrate (IRS) has an important role as a mediator of insulin and IGF-1 signaling. Animal studies revealed that mice lacking IRS had lower bone formation and bone turnover [112]. IRS-1 knockout mice have impaired bone healing, and IRS-2 knockout mice had decreased bone formation and increased bone resorption [113].

The indirect effects of insulin on bone are accomplished by blood glucose control. Calcium metabolism plays a fundamental role in bone metabolism. Calcium secretion was higher in diabetic rat kidneys than in normal rats [114]. And there is a correlation between urinary calcium production and reduced bone mineral density in diabetic mice [115]. At the same time, hyperglycemia increases the excretion of magnesium and phosphorus, also reduces the renal tubular reabsorption of magnesium and phosphorus, then showed a negative calcium balance and an increased secretion of PTH, leading to decreased bone mineral density and osteoporosis. Animal experiments have demonstrated that bone has an insulin receptor. Stimulation of osteoblasts with exogenous insulin can increase glucose uptake [116]. Insulin control of blood sugar can reduce the excretion of calcium, magnesium, and phosphorus, thus preventing the occurrence of osteoporosis.

Vascular complications of diabetes are also a cause of osteoporosis. An animal study showed that type 1 diabetes can cause abnormal changes in the vascular system, represented by type H vessels in bone, which can uncouple the production of blood vessels and bone production, decreased bone mass, and increased bone fragility. However, the early adequate application of insulin can control blood glucose levels, increase the number of H-type vascular endothelial cells, and avoid the inhibition of the osteogenesis process or reduce the bone mass [117].

But existing studies do not entirely believe that insulin is beneficial for osteogenesis. Zhang et al. suggested that insulin could not only inhibit the autophagic process of BMSCs but also promote cellular senescence, eventually leading to the inhibition of osteogenesis [118]. Transforming growth factor *β*1 could facilitate the above process. Insulin most likely activates the transforming growth factor *β*1 signaling pathway by increasing the expression of the transforming growth factor *β*1 receptor II. And inhibition of transforming growth factor *β*1 signaling prevents the negative effects of insulin on BMSCs. The longitudinal study by Ruppert et al. also suggested that insulin treatment in type 2 diabetes patients had detrimental effects on bone, leading to a decrease in the femoral neck BMD [119].

Insulin use is associated with the incidence of hypoglycemic events in diabetic patients, and more hypoglycemic events also increase the risk of falls, which may also be responsible for the increased risk of fractures in diabetic patients [120, 121]. Related studies also confirm the hypothesis that applying insulin therapy increases the risk of fracture [122]. Patients with type 2 diabetes are treated with insulin when oral hypoglycemic drugs are ineffective. At this time, the patient's disease course may be longer, and it is difficult to control the blood sugar well. Diabetic complications such as retinopathy and peripheral neuropathy may occur. The risk of a fracture was also elevated [3].

### 3.4. Other Hypoglycemic Drugs

SGLT 2 inhibitors are a new type of hypoglycemic drug. It has been suggested that SGLT 2 inhibitors may exacerbate the loss of trabecular bone [123]. Although the mechanism of this process is not clearly defined, this may be related to its mechanism of glucose reduction. SGLT 2 inhibitors reduce the renal reabsorption of blood glucose while reducing sodium transport. This process increases phosphate cotransport, stimulates the parathyroid gland, and increases bone resorption [124]. Serum phosphate and PTH were both elevated after dapagliflozin treatment [125]. However, some studies also showed that PTH and BMD did not change significantly after dapagliflozin treatment [126]. Therefore, clarifying the effect and mechanism of SGLT 2 inhibitors on bone is beneficial for clinicians to protect the bones of diabetic patients.

Incretins are also a class of hypoglycemic drugs. Glucagon-like peptide 1 (GLP-1) agonists and inhibitors of dipeptidyl peptidase 4 (DPP-4) are both of these drugs. The GLP-1 receptor is expressed in pancreatic islet *β*-cells [121]. It has been suggested that GLP-1 controls bone resorption through GLP-2 and glucose-dependent insulinotropic polypeptide (GIP). In addition to promoting insulin secretion, GLP-1 and GIP also regulate bone turnover and bone homeostasis. GLP-1 receptor knockout mice have lower levels of calcitonin mRNA transcription and higher levels of urinary deoxypyridinoline. The application of calcitonin can effectively improve the above two conditions [127]. Thus, GLP-1 most likely affects the bone resorption process through the calcitonin pathway. However, the results of applying GLP-1 agonists on fracture risk in different clinical studies are contradictory [128, 129]. Most of the studies on DDP-4 inhibitors are clinical studies. Whether the effects of DPP-4 inhibitors on bone are beneficial remains unclear. However, DPP-4 inhibitors are less harmful to BMD than thiazolidinediones [130]. Therefore, further studies on the effects of incretin on bone are still needed.

## 4. Fracture Risk in Diabetic Patients

Patients with diabetes have a higher incidence of osteoporosis and a higher incidence of fragility fractures than nondiabetic patients. For these patients, complications of diabetes such as neuropathy, retinopathy, and occasional hypoglycemia can lead to an increased risk of falls or even fractures [131]. Some possible mechanisms have also been proposed to explain this increased fracture risk in diabetic patients, such as an increased risk of falling, microvasculopathy, and the accumulation of glycation end products [132–135]. Among them, the bone characteristics of type 1 diabetes mellitus and type 2 diabetes mellitus patients are also different again. Studies have reported that patients with type 1 diabetes have lower bone mineral density compared with nondiabetic patients, which may be responsible for the higher risk of fracture in this population. However, BMD in type 2 diabetic patients has different results compared with nondiabetic patients, with some studies reporting lower BMD in type 2 diabetic patients, while some studies considered it to be similar or higher [136, 137]. This suggests that BMD, or rather in patients with type 2 diabetes, is not a major contributor to fracture risk. Whether in type 1 diabetes or type 2 diabetes, the resulting bone microstructural changes reduce bone mass and also lead to an increased fracture risk. In addition, it has been shown that certain medications for diabetes are also risk factors for fragility fractures [138].

The incidence of hip fracture is significantly increased in type 2 diabetic patients compared with nondiabetic patients [139, 140]. Some studies believe that BMI is a protective factor for the fracture development in diabetic patients [141]. However, some studies have shown that patients with type 2 diabetes have an increased risk of nonvertebral fractures with a BMI of 24 kg/m^2^ [142]. Furthermore, different studies have shown a reduced prevalence of vertebral fractures in type 2 diabetes, with the analysis that is due to older age or the presence of obesity [143].

Another possible reason contributing to the increased risk of fracture in patients with type 2 diabetes is its lower bone turnover status. In the cortical bone, low bone turnover may lead to the occurrence of microcracks, so that the risk of nonvertebral fractures is increased [144]. The results observed by Hygum et al. suggest that the bone resorption marker c-terminal crosslinked terminal peptide (CTX) and the bone formation markers osteocalcin and procollagen type 1 are significantly reduced in people with type 2 diabetes, which also supports the above speculation of low bone turnover status [145].

## 5. Assessment of the Fracture Risk in Patients with Diabetes Mellitus

In clinical practice, double-energy X-ray analysis is often used to assess bone mineral density, and the fracture risk is assessed by using the fracture risk assessment tool (FRAX). Generally, people with lower BMD will have a higher risk of fracture than those with normal BMD, so for the general population, FRAX can be used to understand the risk of fracture. However, using FRAX to assess the fracture risk in patients with type 2 diabetes is not accurate enough.

Considering that in patients with type 2 diabetes, bone microstructure is damaged due to the accumulation of AGEs, and conventional FARX cannot predict the fracture risk caused by this pathological change well; a fracture risk prediction tool for type 2 diabetes patients is needed to evaluate the fracture risk in this population, so as to conduct timely intervention. Gao et al. try to optimize FARX, an optimization before the use of conventional FARX for type 2 diabetes and nondiabetes fracture risk prediction, and found that type 2 diabetes patients have lower fracture risk; this is inconsistent with most of the results of the study, and by adding rheumatoid arthritis as an equivalent variable, it can be clearly observed that type 2 patients with a fracture have a higher risk of diabetes than nondiabetes patients [146]. In other studies, even if FRAX was adjusted with rheumatoid arthritis, the results were still different from those actually observed [147]. Although the study of Gao et al. has some directions for attention, its sample size is still limited, and a large number of samples is still needed to further improve this assessment method.

Some studies suggest that the decline in trabecular bone score (TBS) is associated with type 2 diabetes mellitus [148, 149]. There are studies that also suggested that the lumbar spine trabecular score is a potential tool for fracture risk prediction beyond BMD [150]. A meta-analysis showed that TBS were significantly lower in diabetic patients compared with nondiabetic patients [151]. Another study showed a negative correlation between TBS and fasting blood glucose level [149]. However, TBS currently only evaluates the bone microstructure of the lumbar spine, and the distal radius or the hip can not be well evaluated by this method [152]. In addition, most of the research subjects to predict fracture risk through TBS are postmenopausal women, and there are few related studies in other populations [153]. Therefore, the specific role of TBS on fracture risk prediction in diabetic patients is still unknown, and the correlation between TBS and the criteria for the fracture prediction in diabetic patients is still needed.

Unlike TBS, quantitative computed tomography (QCT) measures the real volume of bone mineral density (vBMD). The vBMD is better able to detect changes in bone strength than that measured by the dual-energy X-ray. High-resolution peripheral quantitative computed tomography (HR-pQCT) allows the imaging of bone microstructures, including cortical porosity [154]. However, HR-pQCT is mostly used to measure the distal radius and distal tibia rather than the lumbar spine [155]. A study of patients with type 2 diabetes showed clear differences in cortical porosity between type 2 diabetes and nontype 2 diabetes [156]. Therefore, HR-pQCT has the potential to be a fracture risk prediction tool in diabetic patients.

Both TBS and HR-pQCT are assessed for fracture risk by imaging. Diabetic patients are in a low bone turnover state, so that serum bone turnover markers may play a positive role in fracture risk prediction. The dynamic balance between bone formation and bone resorption is destroyed in the case of long-term hyperglycemia. Studies have found that the serum osteocalcin in diabetic patients is significantly lower than that in people with normal blood glucose, and the serum osteocalcin level continues to decrease from the increase of abnormal blood glucose to the diagnosis of diabetes [157]. Internationally, the c-terminal crosslinked terminal peptide (CTX) of collagen I and the n-terminal propeptide (P1NP) of collagen I are recommended as a reference marker for monitoring bone turnover [158]. Ivaska et al.'s study of 75-90 years showed that high levels of CTX are associated with a higher risk of fracture in 75 years. P1NP may be more valuable for young osteoporosis patients, while predictive assessment of fracture risk by bone turnover markers seems to lose value when patients exceed 80 years [159]. Some studies have shown that osteocalcin, CTX, and P1NP are lower in diabetic patients than in nondiabetic patients [145]. Additional studies showed that increased CTX and P1NP levels in nondiabetic patients were associated with an elevated fracture risk, but such an association was not observed in diabetic patients [160]. In addition, pentosidine can also be used as a biochemical marker to predict fracture risk. The pentosidine content in the cortical bone of trabecular bone, femur, and tibia is negatively correlated with the ultimate stress and toughness of bone [161]. Even among the nondiabetic populations, the pentosidine level is higher in fracture patients [162]. This indicates that the increase of pentosidine is one of the manifestations of bone deterioration. And pentosidine can be used as a predictor of fracture risk. The secretory RAGE is a variant of RAGE. Lower levels of secretory RAGE were found to be associated with a higher risk of vertebral fractures [163]. Another study suggested that secretory RAGE was not associated with fracture risk in the type 1 diabetes population [164]. This may imply that the effect of AGE-RAGE signaling on bone fragility is only manifested in patients with type 2 diabetes. Activation of the Wnt/*β*-catenin signaling pathway is an important process of osteoblast differentiation. As a Wnt inhibitor, sclerostin can negatively regulate this process [165]. Animal studies showed that the bone mass increased in all parts of osteoporosis rats treated with sclerostin antibody [166]. Thus, it is possible that sclerostin levels may also be used as biomarkers to assess bone mass and even indirectly predict fracture risk. Therefore, the risk predictive ability of bone turnover markers for osteoporotic fractures in diabetic patients still needs further study and confirmation.

## 6. Conclusions and Prospects

In conclusion, patients with diabetes are a high-risk group for osteoporosis. The mechanism between diabetes and osteoporosis is not fully defined. Ferroptosis and sex hormones may be important routes for osteoporosis in diabetic patients. Defining their mechanism of action may benefit the prevention and treatment of osteoporosis in diabetic patients. Most of the studies on the effects of traditional and new hypoglycemic drugs on bone are retrospective studies. The mechanisms by which these drugs affect bone require further investigation. The impact of various diabetes treatment drugs after long-term use on bone needs to be improved urgently. At present, there is no gold standard for the assessment of fracture risk in diabetic patients, and there are some deficiencies in various types of auxiliary examinations. According on the characteristics of osteoporosis in diabetic patients, further improving the inclusion criteria for FRAX risk factors may be an effective risk assessment tool for osteoporotic fractures in diabetic patients. TBS and HR-pQCT need to break through technical barriers to add more accurate and effective measurement locations. Identifying the site of early osteoporosis in diabetic patients may facilitate the development or improvement of assessment tools for the diagnosis of diabetic osteoporosis.
